# Exploring the Origin and Antigenic Specificity of Maternal Regulatory T Cells in Pregnancy

**DOI:** 10.3389/fimmu.2020.01302

**Published:** 2020-06-25

**Authors:** Soo Hyun Ahn, Sean L. Nguyen, Margaret G. Petroff

**Affiliations:** ^1^Department of Pathobiology Diagnostic Investigation, College of Veterinary Medicine, Michigan State University, East Lansing, MI, United States; ^2^Institute for Integrative Toxicology, Michigan State University, East Lansing, MI, United States; ^3^Cell and Molecular Biology Program, Michigan State University, East Lansing, MI, United States; ^4^Department of Microbiology and Molecular Genetics, Michigan State University, East Lansing, MI, United States

**Keywords:** central tolerance, peripheral tolerance, Aire, thymic T_Regs_ peripheral T_Regs_, fetal antigens, paternal antigens, pregnancy

## Abstract

Successful pregnancy outcome is partially determined by the suppression of reactive effector T cells by maternal regulatory T cells (T_Regs_) at the maternal-fetal interface. While a large area of research has focused on the regulation of peripherally-induced T_Reg_ (pT_Reg_) distribution and differentiation using transgenic mouse models and human samples, studies focusing on the role of T_Regs_ derived from the thymus (tT_Regs_), and the potential role of central tolerance in maternal-fetal tolerance is less explored. The genome of the fetus is composed of both the tissue-specific and paternally-inherited antigens, and a break in maternal immune tolerance to either antigen may result in adverse pregnancy outcomes. Notably, “self”-antigens, including antigens that are highly restricted to the fetus and placenta, are promiscuously expressed by medullary thymic epithelial cells under the control of Autoimmune Regulator (Aire), which skews the tT_Reg_ T cell receptor (TCR) repertoire to be specific toward these antigens. T_Regs_ that circulate in mothers during pregnancy may be comprised of T_Regs_ that stem from the thymus as well as those induced in the periphery. Moreover, despite a wealth of research dedicated to elucidating the function of T_Regs_ in maternal-fetal tolerance, little is understood about the origin of these cells, and whether/how tT_Regs_ may contribute. Investigation into this question is complicated by the absence of reliable markers to distinguish between the two. In this review, we discuss how distinct types of fetal/placental antigens may determine the generation of different subtypes of T_Reg_ cells in the mother, and in turn how these may promote maternal tolerance to the fetus in pregnancy.

## Regulatory T Cells Are Critical Players in Pregnancy Outcome

The immunological paradox of the semi-allogeneic fetus has been a key question in reproductive physiology and immunology for decades. There are few examples in which one molecule or cell type can account for maternal tolerance to the fetus; an exception is CD4+CD25+Foxp3+ regulatory T (T_Reg_) cells. T_Reg_ cells were identified after years of controversy based on their expression of the IL2α receptor, CD25, and their critical role in protection against autoimmune disease (1). Many studies in mice have demonstrated the indispensable role of CD4+CD25+Foxp3+ T_Regs_ for maintaining immune homeostasis (2); mice lacking functional Foxp3, the critical lineage-determining factor for T_Reg_ cells, develop a phenotype characterized by scaly, ruffled skin, and an enlarged spleen (3–5). In humans, Foxp3 mutations result in IPEX syndrome (immune dysregulation, polyendocrinopathy, enteropathy, X-linked). Both humans and mice lacking functional Foxp3 suffer severe autoimmune disease and are at high risk of early death due to autoimmune disease caused by a deficiency in T_Reg_ cells.

The additional role of T_Reg_ cells in pregnancy was identified in a seminal study that used a depletion/replacement experimental strategy in mice to show that these cells are critical for immunological protection of the semiallogeneic fetus (6, 7). This finding has been independently verified by many laboratories, with the additional finding that T_Reg_ cells are particularly important around the time of implantation (8–11); interestingly, Rowe et al. (12) cautioned that T_Reg_ cells could be detrimental to pregnancy in terms of potentially limiting the ability of mothers to fight infection. Information regarding T_Reg_ cells in human pregnancy has been obtained from peripheral blood and samples obtained at the maternal fetal interface from pregnant women: initial studies compared decidual and peripheral blood T_Reg_ cells in the first trimester of healthy pregnancy and pregnancies complicated by spontaneous abortion (13). The proportion of T_Reg_ cells in the decidua was ~3-fold higher than in peripheral blood, suggesting recruitment and/or *in situ* proliferation of these cells. Moreover, decidual T_Reg_ cells were significantly decreased in spontaneous abortion cases. Further studies of spontaneous abortion and pre-eclampsia have supported the notion that optimal T_Reg_ cell responses are necessary to avoid detrimental pregnancy outcomes in women (14, 15). Collectively, these studies suggest that generation and recruitment of T_Regs_ to the maternal fetal interface are important in protecting optimal survival of the allogeneic fetus, while maintaining the ability of the mother to fight infection during pregnancy.

## Origins of T_Reg_ Cells and Their Cognate Antigens

CD4+CD25+Foxp3+ T_Reg_ cells arise from two overarching mechanisms: during thymocyte development and differentiation in the thymus, or by differentiation of circulating peripheral CD4+ cells following their exit from the thymus. Peripherally induced T_Reg_ (pT_Reg_) result from the conversion of mature circulating conventional CD4+CD25- T cells into T_Reg_ cells in response to low-dose foreign antigens (2). Such is the case in Gut-Associated Lymphoid Tissue (GALT) and lymph nodes (LNs) draining the intestines, where pT_Reg_ cells with T cell receptor (TCR) specific to gut microbiota are found (16). These T_Reg_ develop in response to TCR and TGF-ß signaling through binding of NFAT (Nuclear Factor of Activated T cells) and Smad3 (Mothers against decapentaplegic homolog 3) to the CNS1 (Conserved Noncoding Sequence 1) element in the promoter region of *Foxp3* (17). CNS1 is indispensable for the generation of pT_Regs_: in CNS1-deficient mice, induction of Foxp3 in naïve CD4+ T cells and consequent generation of pT_Reg_ was impaired *in vivo* (18). Importantly, Foxp3 expression is not sustained in pT_Reg_ cells if TGF-ß is removed; thus, stability of Foxp3 expression and functional activity of pT_Reg_ cells are relatively low (2, 19).

The necessity of the CNS1 element was also investigated in pregnancy. It appears logical to expect that pT_Reg_ cells are key in pregnancy success: antigens inherited from the father could be neither present nor expressed by the maternal thymic genome—a key component of thymic T cell tolerance and generation of thymic T_Reg_ (tT_Reg_). Instead, introduction of paternal alloantigens at coitus, and later as conceptus, could induce the generation of pT_Regs_. Intriguingly, the Foxp3 binding site within CNS1 is highly conserved among placental mammals, in which pregnancy involves long, sustained, direct contact between maternal and fetal cells; the binding site was not conserved in non-eutherian mammals (e.g., marsupials) and non-mammals (20). In the same study, expansion of T_Reg_ cells in allogeneically-mated females appeared to be dependent on CNS1, and rates of resorption, albeit relatively low overall, were higher in CNS1-deficient mice in comparison to CNS1-sufficient mice, as well as in comparison to syngeneically-bred controls. This work agrees with that of Rowe et al. who found that adoptively transferred naïve CD4+ T cells with specificity to a surrogate paternally-inherited antigen upregulated Foxp3 expression and gained protective function during pregnancy (21, 22).

Other studies, however, implicate the importance of T_Reg_ generated in the thymus—tT_Reg_–in establishing maternal tolerance to the fetus. These cells commit to the T_Reg_ lineage as early as the CD4+CD8+ double-positive stage of T cell development in a manner dependent on TCR and IL-2 signaling (23). In the thymic medulla, single-positive CD4+ T cells with properly arranged TCRs can develop into Foxp3+ T_Reg_ after encountering self-antigen/MHC II complexes expressed by thymic antigen presenting cells (APC) (24). High affinity/avidity signals from self-antigen/MHC II through the TCR leads to upregulation of CD25 and increased sensitivity to IL-2 (25–28). In contrast to pT_Reg_ cells, in which Foxp3 expression is relatively unstable, Foxp3 expression in tT_Reg_ cells are highly stable due to sustained demethylation of the CNS2 region of the promoter (29–31). For a more detailed comparison between the developmental requirements of tT_Reg_ and pT_Reg_, readers are referred to excellent reviews (2, 32).

The relative Foxp3 stability of tT_Reg_ over pT_Reg_ may have important implications for pregnancy: thymus-derived T_Reg_ cells may offer a distinct functional advantage over pT_Reg_ cells given the length of pregnancy and need for long-lasting tolerance. Further, tT_Regs_ and pT_Regs_ may possess dissimilar TCR repertoires due to the source of and mode of exposure of T cells to fetal/placental antigens. Indeed, one may categorize fetal antigens into two groups: those arising exogenously after inheritance from the father, and tissue-specific antigens. Exogenous fetal/placental antigens include paternally-inherited major and minor histocompatibility antigens that differ between parents (33); these are known to elicit maternal T cell reactivity (34). On the other hand, fetus- and/or placenta-specific antigens may arise not due to their mode of inheritance, but simply as a result of being highly tissue-specific, and as such, perceived as antigenic by the mother.

It is currently unknown whether fetus/placenta-specific antigens promote T_Reg_ cell generation in the mother during pregnancy. However, thymic generation of tissue-specific T_Reg_ cells is critical for tolerance to many tissues. As explained further below, the expression of tissue-specific proteins occurs in the thymus under the control of the transcriptional regulator Aire (Autoimmune Regulator), and leads to both deletion of autoreactive conventional T cells and generation of tissue-specific T_Reg_ cells (35–37). Absence of functional Aire is associated with severely reduced fertility and early pregnancy loss in both mice and in women, and a number of fetus- and placenta-specific antigens are expressed in the thymus in an Aire-dependent manner (38–41). Additionally, tissue-specific antigens may access the thymus via the bloodstream (42); studies using TCR transgenic mice show that fetal antigen may do so (43). The possibility that fetal/placental antigens access or are expressed in the thymus raises the possibility that tT_Reg_ arise as a result, and further, that they possess TCR repertoires distinct from pT_Reg_ cells. Thus, distinct types/sources of antigens may determine the type and relative stability of T_Reg_ cells that are formed and are thus responsible for tolerance to fetal/placental antigens during pregnancy. Whether these cells have additional distinct roles in the local environment (i.e., at the maternal-fetal interface) or systemically is unknown, but this warrants investigation.

## Identification of pTreg And tT_Reg_

Due to their distinct developmental mechanism, potential differences in TCR repertoire, antigen specificity, and differential stability, a mechanism is required to reveal their contribution to maternal-fetal tolerance in pregnancy. To this end, Helios, an ikaros transcription factor family (44), and neuropilin-1 (Nrp-1), a single transmembrane receptor (45, 46), offer tools for distinguishing tT_Reg_ cells from pT_Reg_ cells; however, our ability to do so remains imperfect. Using mouse models, Singh et al. demonstrated higher expression of Helios than Nrp1 in thymic CD4+CD8-CD25+ cells compared to pancreas draining LN and spleen, suggesting that Helios may be superior to Nrp1 to detect tT_Reg_ (47). However, this study did not rule out the possibility that Helios and Nrp1 could be induced in the periphery and thus may also identify T cells that are recirculating back into the thymus. Comparison of TCR repertoires between Helios-sufficient and Helios-deficient T_Reg_ from mesenteric LNs has shown both similarities (48) and differences (49) between the two populations, suggesting that Helios may not be a reliable marker for tT_Reg_. Helios can also be induced in culture on CD4+ T conventional cells by inflammatory stimuli (50–53), and in humans, both Helios+ and Helios- Foxp3+ tT_Reg_ cell populations can be found (54). Collectively, these data suggest that using presence of Helios alone to discriminate between tT_Reg_ and pT_Reg_ is insufficient (32).

Nrp1 is a multifunctional single-pass transmembrane receptor that participates in axonal growth and angiogenesis as well as in immune regulatory functions (55). Nrp1 was shown in early studies to be highly expressed by CD4+CD25+ T cells with suppressive capability, but not on naïve CD4+CD25- T cells (56, 57). Nrp1 functions to increase sensitivity of T_Reg_ to antigen and permit prolonged interaction with immature dendritic cells (57). Further, its expression is associated with prolonged survival of allogeneic grafts in mice (58) as well as demethylation at the CNS2 region of the Foxp3 locus (59), which is characteristic of tT_Regs_. On the other hand, 90% of CD4+CD8+ DP cells in the thymus are Nrp1+ (60) and can also be induced in conventional CD4+ T cells *in vitro* (58). Thus, further studies are required to discern whether expression of Nrp1 is constitutive or inducible in tT_Regs_ *in vivo* and *in vitro*.

Our inability to discriminate tT_Reg_ cells from recirculating pT_Reg_ cells within the thymus has hampered our ability to study these subpopulations. A recent discovery, however, has provided a fresh approach (61). Using CD73, an ectonuclease receptor that converts adenosine monophosphate to adenosine (62), together with Rag2-GFP animals, CD73-expressing cells were found to represent mature peripheral recirculating cells, whereas CD73-negative cells represented newly made thymic T cells. This study also showed that tT_Reg_ progenitors initially possess an immature CD24^hi^Qa-2^lo^ phenotype (61), subsequently progressing to more mature CD24^lo^Qa-2^hi^ profile. Interestingly, one of the first studies to identify Nrp1 as a potential marker for tT_Reg_ showed that the Nrp1^lo^ population in the thymus is of the same immature CD24^hi^Qa-2^lo^ phenotype while the vast majority of Nrp1^hi^ cells represented the more mature CD24^lo^Qa-2^hi^ subset (45). Thus, the findings of these two studies suggest that immature CD73-negative tT_Reg_ progenitors may acquire Nrp1 expression as they mature through progenitor stages in the thymus.

Similarly, using a dual reporter mouse model for Foxp3 and Helios, Thornton et al. simultaneously measured both markers in T cells isolated from mesenteric LN, and found lower expression of CD73 in Helios-deficient T_Regs_ compared to Helios-sufficient T_Regs_ (49)_._ Therefore, while CD73 expression can confidently be used to distinguish between newly generated thymic cells from recirculating peripheral cells, we still lack conclusive markers to differentiate between peripheral and thymic T_Reg_ cells in the peripheral lymphoid organs or peripheral blood (63). It is worthwhile to investigate whether CD73 expression can be gained by tT_Regs_ as they exit the thymus, and whether CD73-negative cells in the thymus express Nrp1 and/or Helios. These findings may provide effective means to distinguish tT_Regs_ and pT_Regs_.

## Could Tolerance to the Fetus be Driven by tT_Reg_ Cells in Pregnancy?

In both mice and women, changing dynamics of T_Reg_ cells are evident early in pregnancy. In women, abundance of T_Reg_ cells in the uterus fluctuates with the menstrual cycle (64), and during pregnancy, T_Reg_ cell accumulation in the first trimester decidua is associated with their decrease in the peripheral blood (65). Similarly, in mice, T_Reg_ cells accumulate in the uterus as early as coitus (9). While these early trafficking patterns suggest that T_Reg_ cells migrate to the maternal-fetal interface from the peripheral circulation, they do not reveal the origin of T_Reg_ cells that accumulate there.

Although there is evidence supporting a role for pT_Regs_ in pregnancy (20), emerging evidence also suggests that tT_Reg_ are important. There are conspicuous changes that occur in the thymus during pregnancy, even as early as the first trimester in the mouse. Under the control of ovarian hormones, the murine thymus gradually declines in size and cellularity, ultimately losing up to 90% of its cells by late gestation (66–68). The thymus normally exports millions of new T cells daily; however, during pregnancy, thymocyte output is reduced (69, 70). Older studies suggest that similar pregnancy-associated reductions in thymic mass also occur in women (63, 71). Loss of thymocytes in mice is accompanied by decreased recruitment of thymocyte progenitors from the bone marrow, loss of proliferative activity by early stage CD4-CD8- double negative thymocytes, and reduced production of chemokines and growth factors regulating immigration, proliferation, and migration of thymocytes and their precursors (68, 70, 72). All populations of CD4+ and CD8+ T cells appear to be similarly affected, including T_Reg_ cells (72). These changes have been postulated to be necessary to diminish the output of effector T cells with fetal/paternal antigen specificity and/or to promote production of lymphocytes beneficial for pregnancy (68, 73). One study showed that thymic involution and pregnancy outcome influence each other; When thymus involution is prevented in dams during pregnancy, the number of viable implantation sites decreased and number of resorbed sites increased (68). Deletion of fetal/paternal antigens in the thymus is possible but to our knowledge, no studies have shown this to be the case. More studies are needed to elucidate the role of the thymus in pregnancy outcome.

One possibility is that the thymic changes during pregnancy result in altered/increased output of highly stable tT_Reg_ cells with antigenic specificity to the fetus. Evidence supporting this is, however, conflicting. In mice, absolute numbers of CD4+CD25hi T cells in the thymus are reduced; however, their relative proportions are maintained or increased (70, 72). In women, Heliburg et al. reported maintained output of tT_Reg_ cells during pregnancy (74), while Wagner et al. found a decrease in circulating recent thymic emigrant (RTE) tT_Reg_ (75). Despite this decrease, however, these authors found increased proliferative activity of RTE-tT_Regs_ with superior suppressive activity compared to more mature T_Reg_ cells. Consistent with this idea, in mice, tT_Reg_ cells as defined by Nrp1 expression and demethylated status of the CNS2, proliferate in the uterus-draining para-aortic LNs as early as GD3.5 (59). The presence of organ-specific antigens can trigger local expansion of organ-specific T_Reg_ cells in draining LNs with considerable inter-organ differences in TCR usage by T_Reg_ (16). As such, tT_Reg_ in uterus-draining LNs are likely responding specifically to fetal/paternal antigens in the para-aortic LNs by proliferation (59). The notion of early recruitment of tT_Reg_ cells to the decidua was also suggested by Teles et al. who demonstrated increased Foxp3+ Helios+ T_Reg_ cells in the thymus, uterus-draining LNs, and uterus by day 2 of murine pregnancy (76). Collectively, these studies in women and in mice support the idea that tT_Reg_ cells may expand and proliferate in the peripheral LNs during pregnancy and may provide strong suppressive activity.

## Is There a Role of Maternal Thymic Aire in Immune Tolerance to Fetal antigens?

An individual's capacity to tolerate self and respond to foreign antigen is determined by the TCR repertoire of conventional CD4+ and CD8+ T cells, and Foxp3+ T_Reg_ cells in the periphery. An important determinant of the CD4+ T cell and T_Reg_ TCR repertoire is Aire, which is expressed primarily by medullary thymic epithelial cells (mTECs) (35) and has an unusual mechanism of action that promotes transcription of genes marked with transcriptional silencers. As a result, Aire induces the expression of a wide range of chromosomally clustered genes that are otherwise restricted to certain tissues (77–79). This expression results in direct or indirect presentation of tissue-restricted antigens in the context of major histocompatibility complex (MHC) molecules by mTEC or resident dendritic cells, respectively (80). Thymocytes with TCR specificity for Aire-regulated self-antigens may then undergo one of two possible fates: negative selection, wherein the autoreactive cells undergo apoptosis, or differentiation into self-antigen-specific tT_Reg_ cells (37, 81, 82).

Aire-mediated tolerance provided by this mechanism is pivotal: in humans, genomic mutations in Aire gene result in a devastating autosomal recessive monogenic disease called Autoimmune Polyglandular Syndrome type 1 (APS-1), with prototypical clinical manifestations that include hypoparathyroidism, primary adrenocortical insufficiency, and chronic mucocutaneous candidiasis (83, 84). Mice lacking Aire vary in phenotype according to their genetic background strain: those on the C57BL/6 background experience only mild autoimmune disease with only few affected organs, whereas those on the SJL/J and NOD backgrounds succumb to fatal disease. Aire-deficient mice on the Balb/c backgrounds experience intermediate disease (85).

Autoimmunity in Aire-deficient mice is at least partially explained by alterations in tT_Reg_ development. Knockout mice show reductions in tT_Reg_ cell numbers (86), and autoimmune disease in neonatal NOD mice lacking Aire can be prevented by donor T_Reg_ from Aire-sufficient mice (87). Malchow et al. demonstrated that T cells infiltrating the prostate in knockouts possessed TCR repertoires preferentially expressed by T_Reg_ cells in Aire-sufficient mice, suggesting that Aire enforces tolerance to self-antigen by driving autoreactive T cells toward the tT_Reg_ lineage (37).

Genes regulated by Aire represent virtually all tissues in the body, including reproductive tissues such as the ovary, uterus, and male reproductive tract. Gene expression profile studies of human and murine TEC reveal that Aire also regulates genes restricted to developmentally important tissues, including the pre- and post-implantation embryo, fetus, and placenta (41, 88). RNA sequencing analysis of mTECs from 6 to 8 week old WT and Aire-deficient mice revealed representation of placental antigens within the top 10 represented organs overall (88). Additionally, Gotter et al. showed that human mTECs express a highly diverse selection of tissue-specific genes colocalized within chromosomal clusters: encompassing autoantigens and placenta-associated antigens including CGA, PRG2, SDC1, SEMA3B, CHS2, and CLDN4 (89). Recently, HLA-G was postulated to be regulated by Aire, consistent with earlier findings that identified a subset of TECs sharing expression of this placenta-specific gene with placental trophoblast cells (90, 91). Therefore, it is possible that these antigens, when expressed by mTEC, help shape the mature T cell repertoire, including that of tT_Regs_ that are recruited to the maternal-fetal interface and uterus-draining LN to monitor and provide tolerance to the developing fetus.

## Sources of T_Reg_ Stimulation in Pregnancy

Most studies in mice agree that both syngeneic and allogeneic pregnancies, in which the dams and sire are of identical or dissimilar genotypes, respectively, involve early and local expansion of T_Reg_ cells, with an earlier and higher degree of expansion occurring in allogeneic pregnancy (6, 22, 92, 93). These responses may be mediated by multiple stimuli, including endocrine changes and/or “foreign” antigens that are inherited from the father. A recent study suggested that extravillous trophoblast cells can induce Foxp3 expression in naïve CD4+ T cells via a contact-independent mechanism, suggesting non-antigenic stimulation and further, that these cells may not be specific for fetal antigen (94). Fetal trophoblast cells that lack MHC do not have the ability to present antigen to T cells directly; further, since all trophoblast cells lack MHC class II, it would seem that they would be incapable of directly stimulating CD4+ T_Reg_ cells. In agreement with this, mice lacking MHC revealed that presentation of fetal antigens *in vivo* is predominantly or entirely accomplished through cross presentation by maternal antigen presenting cells (APCs) (42, 95).

Paternally-inherited antigens provided by the fetus to which the mother may react can include major and minor histocompatibility antigens (89). This has been confirmed in human pregnancy: minor antigen-specific T cells, as well as antibodies to male antigens, can be identified in multiparous women (96, 97). Further, maternal reactivity to paternally-inherited antigens appears to be of clinical significance: in hematopoietic cell transplant cases in humans, male recipients of cells from parous females have increased risk of graft vs. host disease, possibly due to co-transferred anti-male antigen specific T cells resulting from prior pregnancy (97).

Evidence for antigen specificity of pT_Reg_ cells was provided by Rowe et al. which showed that naïve CD4+ cells with paternal-antigen specificity became Foxp3+ after transfer into T_Reg_-deficient hosts (19). Schumacher et al. used the abortion prone CBA/J x DBA/2J model to show that minor histocompatibility antigens are likely key to induce maternal CD4+CD25+ T_Reg_ cells that promote pregnancy success: whereas DBA/2J and Balb/c sires possess the same major histocompatibility haplotype, they likely differ in a number of minor histocompatibility antigens—yet pregnancy is compromised with only the DBA/2J sires and can be rescued by pre-treatment of the dams with T_Regs_ obtained from CBA/J x Balb/c matings (98).

While pT_Reg_ cells play a role in maternal tolerance to the fetus (20, 21), a possible role of central tolerance in pregnancy is less well understood and explored. Fetal/placental antigens may access the thymus via the vasculature; using a surrogate antigen, we showed evidence that this may occur. Fetal antigens may be able to access the thymus, thereby inducing T_Reg_ at that location (43), in the same way that the central nervous system protein myelin basic protein induces negative selection of CD4+ T cells despite its lack of synthesis in the thymus (99). Alternatively, tissue restricted antigens that are expressed in the thymus, including those regulated by Aire, may induce tT_Reg_ development. Indeed, Aire-deficient mice have impaired fertility, and while this is associated with autoimmune-mediated oophoritis, we recently showed that peri-embryonic loss can occur as well (39). Further studies will determine whether Aire-mediated central tolerance via tT_Reg_ generation is necessary for maternal tolerance to the fetus.

## Conclusion

**Figure 1:**
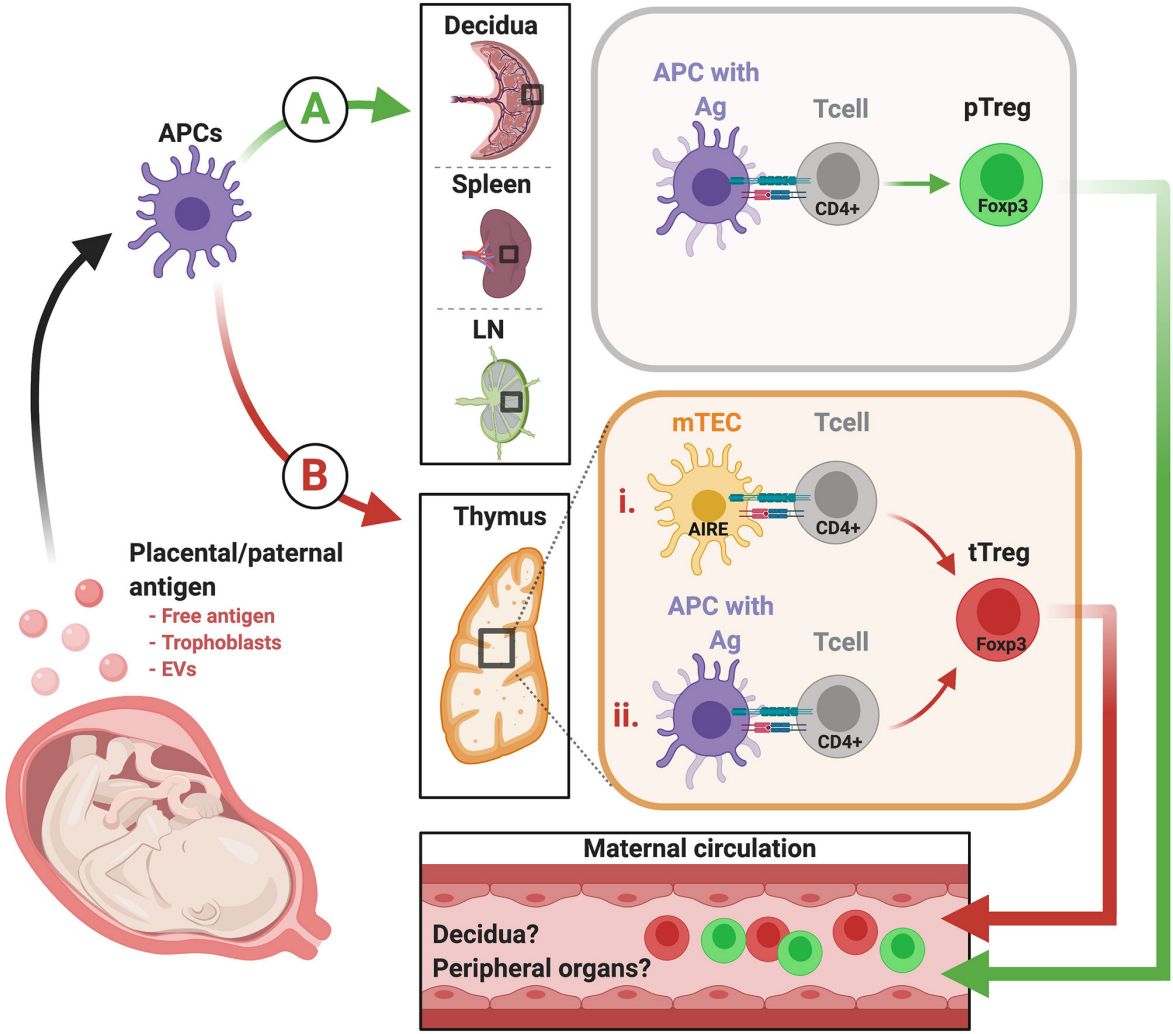
Proposed model of how fetus-specific and paternally-inherited antigens can contribute to pT_Reg_ and tT_Reg_ development. Fetal and placental antigens that elicit maternal T cell reactivity include those that are paternally-inherited and thus foreign to the maternal immune system, and may also include antigens restricted to fetal and placental tissues. These antigens may arise from chorionic villi, extravillous trophoblast cells, or both, and can be released in the form of whole cells (microchimerism), EVs, and/or as free soluble antigen. From there, placental antigens may prompt T_Reg_ development in either or both of two possible pathways. **(A)** Placental antigens may be presented by maternal APCs in the decidua, and/or drain into maternal blood and lymphatic vessels to access the spleen and uterus-draining LN. At these sites, they can be processed and indirectly presented in the context of maternal MHC to elicit fetal antigen-specific pT_Reg_. **(B)** It is also possible that tT_Reg_ are elicited in the thymus via two potential mechanisms. (i) Placenta/fetus-specific antigens may be expressed and presented directly by mTECs under the control of Aire. (ii) Alternatively, these antigens, and/or placentally-derived antigens accessing the thymus via the vasculature, may be indirectly presented via resident APC in the thymus. EVs, extracellular vesicles; APC, antigen presenting cells; LN, lymph nodes; MHC, major histocompatibility complex; pT_Reg_, peripherally-induced T_Reg_; tT_Reg_, thymus-derived T_Reg_. Created with BioRender.

The fetus is a semi-allograft wherein half of the genome is self and the other half, non-self. Therefore, we must consider the possibility that the suppressive maternal immune component that ensures pregnancy success stems from both tT_Reg_ cells, which may tolerize toward paternally-inherited antigens, fetus/placenta-specific antigens, or both, and pT_Reg_ cells, which may tolerize toward paternally-inherited antigen (Figure 1). Exposure of antigen to maternal T cells likely occurs via release of placental and fetal cells, vesicles, and free protein into the maternal circulation and lymphatics. Priming of naïve maternal conventional T cells and induction/differentiation of T_Reg_ cells could occur locally within the decidua, draining LN, and in thymus; alternatively, expression of placenta- and fetus-specific antigens within the thymus could also mediate de novo generation of tT_Reg_ cells.

It is clear in both mice and women that T_Reg_ cells are required and critically necessary for successful, uncomplicated pregnancy outcome. However, future studies are required to dissect the heterogeneity and antigen specificity of these cells, and the contribution of central tolerance to pregnancy. Further requirements will include identification of clear markers that distinguish between tT_Reg_ and pT_Reg_ by using novel thymus-specific identifiers such as CD73. These tools can then be used to our advantage to finally understand the makeup of T_Reg_ population in pregnancy and their overall contribution to the success of pregnancy outcome in both mice and women.

## Author Contributions

SA and MP conceptualized and wrote the article. SN provided editorial assistance and conceptualized the figure. All authors contributed to the article and approved the submitted version.

## Conflict of Interest

The authors declare that the research was conducted in the absence of any commercial or financial relationships that could be construed as a potential conflict of interest.
